# Relations Between Mood States and Eating Behavior During COVID-19 Pandemic in a Sample of Italian College Students

**DOI:** 10.3389/fpsyg.2021.684195

**Published:** 2021-07-21

**Authors:** Concetta De Pasquale, Federica Sciacca, Daniela Conti, Maria Luisa Pistorio, Zira Hichy, Rosa Loredana Cardullo, Santo Di Nuovo

**Affiliations:** ^1^Vascular Surgery and Organ Transplant Unit, Department of Educational Sciences, University of Catania, Catania, Italy; ^2^Vascular Surgery and Medical-Surgical Specialties, Department of Educational Sciences, University Hospital of Catania, Catania, Italy; ^3^College of Business, Technology and Engineering, Sheffield Hallam University, Sheffield, United Kingdom; ^4^Department of General Surgery and Medical-Specialties, University Hospital of Catania, Catania, Italy

**Keywords:** mood profile, eating disorder, college students, gender differences, fear of COVID-19

## Abstract

The fear of contagion during the COVID-19 pandemic has been indicated as a relevant cause of psychological pathologies occurring in this period. Food represents a compensating experience, distracting from the experiences of uncertainty, fear and despair, causing alterations in eating habits and behaviors. The study aims at evaluating the relations between fear of a pandemic, mood states and eating disorders in Italian college students, taking into account gender differences. During the lockdown for the pandemic, a sample of 469 college students equally distributed by gender, was recruited online using a questionnaire including the FCV-19S for the assessment of fear of COVID-19, the profile of mood states (POMS) for the evaluation of different emotional states, the eating disorder inventory-2 (EDI-2) and the binge eating scale (BES) to evaluate the presence of the levels of eating disorders. As expected, all emotive states measured by POMS (tension, depression, anger, tiredness, confusion) resulted significantly correlated with the fear of COVID-19. Women were more exposed to fear of COVID-19 showing greater tension, fatigue, depression and confusion, and a significantly higher total mood disturbance score than males. Regarding the EDI-2 and BES variables, tension and anxiety resulted significantly correlated also with bulimic behavior, while depression with interoceptive awareness, impulsivity, and binge eating behaviors, without gender differences. In conclusion, the negative impact of the fear of COVID-19 on the emotional profile and eating behavior suggests the need to implement strategies against psychological distress during the pandemic emergency, and to design psycho-educational interventions aimed at modifying the lifestyle for preventing risks of mental disorders fostering health-oriented behaviors.

## Introduction

As of March 2020, coronavirus disease SARS-CoV-2 (COVID-19) has been declared a “pandemic” by the world health organization (WHO). This has led to the need for governments around the world to implement restrictive containment and isolation measures to stem the spread of the virus; these measures have provided for social distancing, isolation and quarantine, shifting mainly online social relations, and educational processes.

The consequences on mental health from the COVID-19 pandemic, because of the containment measures adopted, are comparable to the psychopathology pictures described in similar epidemic situations. Recent reviews of epidemiological studies related to the 2003 SARS epidemic highlighted the presence of post-traumatic symptoms, depression, stress, irritability, anxiety, insomnia, anger and emotional exhaustion, due to the impact of containment measures on mental health (Brooks et al., 2020; Mengin et al., 2020). These syndromic pictures appear almost confirmed by studies on the new pandemic carried out in different parts of the world. A study by Jungmann and Witthöft (2020) showed an increase in virus-related anxiety, especially in subjects with higher health anxiety traits. Furthermore, in a sample of 256 adults Lee and Crunk (2020) showed that hypochondria was among the fear factors that predict pandemic-related psychopathology.

Results of another study (Wang et al., 2020), carried out with 1210 respondents from 194 cities in China and conducted in the early stages of the epidemic, found that 53.8% of respondents rated the psychological impact of the epidemic as moderate or severe; 16.5% reported moderate to severe depressive symptoms; 28.8% reported moderate to severe anxiety symptoms, and 8.1% reported moderate to severe stress levels. Most of the respondents spent 20–24 h a day at home (84.7%); they were concerned about their family members who contracted COVID-19 (75.2%) and were satisfied with the amount of health information available (75.1%). Female gender, student status, specific physical symptoms (e.g., myalgia, dizziness), and poor health were significantly associated with a greater psychological impact of the epidemic and higher levels of stress, anxiety, and depression (Wang et al., 2020).

The literature on eating disorders has affirmed that these have a significant prevalence in women compared to men (McDonald and Thompson, 1992; Striegel-Moore et al., 2009). Although recent research has shown that prevalence in men was previously underestimated, these disorders have a clear female preponderance (Andersen and Yager, 2009). More recently, the study by Yu et al. (2018) analyzed the sexual differences in eating disorders among college students, highlighting that the female gender was a predictor of eating disorders and food addiction. The research on university students by Sonneville and Lipson (2018) showed that women, more than men, are diagnosed and treated for the symptoms of eating disorders and perceive, more than men, a need for treatment. Conversely, men are less likely to recognize that they have symptoms related to eating disorders and are therefore diagnosed and treated less than women.

An Italian study showed a high rate of post-traumatic stress symptoms, depression, anxiety, insomnia, perceived stress and adjustment disorder symptoms, with higher probabilities in young women. The duration of the quarantine, the fear of being infected, the frustration and boredom, related to the reduction or suspension of the daily activities in which everyone was engaged, the fear of not having enough supplies or possibility of supply were the causes of greater psychological stress, as well as the confusion surrounding the often inadequate information on the pandemic (Rossi et al., 2020).

At the beginning of the lockdown an interesting feature highlighted in various studies with an Italian sample, concerned the observation of abnormal and irrational behaviors such as the purchase and accumulation of many basic foods (e.g., sugar, wheat, milk, yeast) and the alteration of eating habits, given the growing flow of news on food uncertainty (Touyz et al., 2020). The most recognized causes of changes in eating habits with the increase in restrictive diets due to the concern related to weight and shape, and/or phases of binge eating seem to be: greater sedentary lifestyle, restrictions on outdoor activities, reduction of physical exercise, alterations in the sleep-wake rhythm and fear of contagion (Fernández-Aranda et al., 2020; Rodgers et al., 2020).

Furthermore, there is an extensive literature identifying that depression is associated during the isolation with an increased risk of overeating (Baenas et al., 2020; Mengin et al., 2020). Recent studies have confirmed the relationship between depressed mood and “pathological” eating habits, depression would be associated with an increased risk of overeating (Trojanowski and Fischer, 2018; Mills et al., 2019, 2020).

The research conducted by Termorshuizen et al. (2020) showed that during the COVID-19 pandemic and the consequent lockdown, the subjects with anorexia nervosa had increased the restrictions, as well as the subjects with bulimia nervosa and/or with binge eating disorder, had increased the binges. However, the same subjects had noticed the positive effects of staying in the family, of having more time for themselves, and were more motivated to stay in the hospital (Termorshuizen et al., 2020).

Regarding the effect of COVID-19 on patients with eating disorders, the severity of the symptoms of these disorders may increase unless a treatment according to guidelines can be started (Peckmezian and Paxton, 2020). The relation between eating behaviors and mood in persons without overt pathologies remains less known.

A particular population affected by mood and eating problems are university students. This population experienced during the pandemic a profound change in attitudes and behavioral habits, due to the shift in distance learning and the need, for many off-site students, to return to the family and the place of origin. With the consequence that they had to move from the university residences of the city center and the lifestyle acquired during the attendance of the courses. This change in behavioral habits could have different influences on the two genders.

In Italy, eating disorders affect more than three million people, of which 95.9% are women and 4.1% are men. The greater vulnerability found in females, adolescence or young adulthood, seems to indicate that these disorders are linked to difficulties in the transition from childhood to adult life. These appear to be triggered by specific physical and hormonal changes characteristic of puberty in females. However, epidemiological research also shows an increase in the male population.

The recent study by Guerdjikova et al. (2019) showed that unlike other eating disorders, the women/men ratio in the binge eating disorder (BED) is more balanced. BED occurs in conjunction with other psychiatric disorders, most commonly mood disorders and anxiety. BED is also associated with obesity and its many complications. Although BED is similar in men and women in treatment presentation and outcomes, some key neurobiological differences should be considered when personalizing treatment.

Understanding sexual differences is a crucial aspect of the prevention and treatment of diet-related disorders.

Recently, various studies have investigated the relationship between COVID-19 and eating habits. Specifically, Amatori et al. (2020) examined a group of 176 college students and highlighted the negative impact of COVID-19 on mood states and healthy eating habits. Huber et al. (2020) found that the COVID-19 pandemic lockdown significantly affected eating habits in 1964 college students. The authors concluded that further investigation is needed to evaluate comorbidities and long-term effects on weight change.

### Aims and Hypotheses

The aim of the present study was to investigate, in a sample of Italian college students, the relations between mood states and eating behavior during the current COVID-19 pandemic, deepening also the analysis of gender differences. Specifically, the main hypothesis is that negative emotive states could be correlated with the fear of COVID-19, prevalently in women. High scores in anxiety and depression are expected to be significantly correlated with a tendency to bulimic behavior. Furthermore, it is hypothesized that the gender variable moderates the impact of COVID-19 on mood states and eating behaviors.

## Materials and Methods

### Participants

A sample of 469 college students (*N* = 221 males, *N* = 248 females) aged between 18 and 34 years (*M* = 22.47; *SD* = 2.70) were recruited via an online questionnaire, using the google form platform. The questionnaire was sent to students of the University of Catania (Italy) and distributed from March 2020 to February 2021. Specifically, only the questionnaires received during the periods and areas of lockdown were used for the purposes of the research. The survey was also shared on the Facebook page of the Department of Educational Sciences. All volunteer participants provided online informed consent and answered the questionnaire anonymously. The students involved in the research lived mainly with families.

This study was approved by the Ethic Committee of the Department of Educational Sciences of the University of Catania (Italy).

### Measures

The participants were given an online protocol that included the Fear of COVID-19 scale (FCV-19S) for the evaluation of fear, the Profile of mood states (POMS) for the assessment of emotional states, the Eating disorder inventory-2 (EDI-2) and the Binge eating scale (BES) to evaluate the presence of eating disorders (APA, 2013).

#### Fear of COVID-19 Scale

The Fear of COVID-19 scale (FCV-19S) (Ahorsu et al., 2020) is a one-dimensional questionnaire composed of seven items (e.g., “I’m very afraid of coronavirus-19”; “It makes me uncomfortable to think about coronavirus-19”; “I can’t sleep because I worry about getting coronavirus-19”), with a 5-point response scale (1 = strongly disagree to 5 = strongly agree) which assesses fear of COVID-19 and its consequences. The score is obtained by adding the scores to the questions. The scale showed good reliability (*α* = 0.87).

#### Profile of Mood States (POMS)

The POMS (Grove and Prapavessis, 1992) is a widespread instrument that measures mood and identifies problematic affective states. The measure is a self-report questionnaire, and it is mainly used in the context of clinical psychology, psychotherapy, and medicine. The questionnaire consists of 58 adjectives that define six mood states: tension-anxiety (T), which describes an increase in somatic tension that may not be observable from the outside or may concern visible psychomotor manifestations; depression (D), which indicates a state of depression accompanied by a sense of personal inadequacy, the uselessness of effort, a sense of emotional isolation, melancholy and guilt; aggression-anger (A), which describes anger and dislike toward others; vigor-activity (V), adjectives that suggest exuberance, energy, euphoria, and optimism; tiredness-indolence (TI), which represents boredom, low energy, and physical fatigue; confusion (C), characterized by a sense of disturbance and linked to the organization-disorganization dimension, anxiety and the feeling of cognitive inefficiency.

The intensity with which a mood is experienced is measured on a 5-point Likert scale (0 = not at all, 1 = a little bit, 2 = moderately, 3 = quite a bit, and 4 = extremely). A TMD can be calculated by adding the scores for tension, depression, anger, tiredness, confusion and then subtracting the score for vigor. The scoring range goes from 0 to 250 ± 40 for the total scale (TMD), from 0 to 36 for the tension scale, from 0 to 60 for the depression scale, from 0 to 48 for the anger scale, from 0 to 32 for the strength scale, from 0 to 28 for the fatigue scale, and from 0 to 28 for the confusion scale. In this study, POMS showed good reliability (*α* = 0.85). Also POMS subscales T, D, A, V, S, and C showed good reliability (*α* = 0.89; *α* = 0.94; *α* = 0.71; *α* = 0.69; *α* = 0.62; *α* = 0.77).

#### Eating Disorder Inventory-2 (EDI-2)

The EDI-2 (Garner, 1991; Espelage et al., 2003; Waldherr et al., 2008; De Pasquale et al., 2013) has been used for the clinical evaluation of the social and behavioral aspects associated with eating disorders. It is also used as a screening tool in non-clinical populations. Specifically, it consists of 11 subscales, for a total of 91 items, where only two scales directly measure eating behavior: drive to thinness (DT), and bulimia (B); the other scales concern the relationship with the body, experiences of inadequacy and other more specifically social areas: body dissatisfaction (BD), ineffectiveness (I), interoceptive awareness (IA), perfectionism (P), interpersonal distrust (ID), maturity fears (MF), asceticism (A), impulsivity (I), and social insecurity (SI). The score for each question is given on a 6-point Likert scale (0 = never, to 6 = always). Higher scores indicated the presence of dysfunctional eating behaviors. In this study EDI-2 showed good reliability (*α* = 0.89). Also, the subscales of EDI-2 showed good reliability (*α* ranging from 0.68 to 0.95).

#### Binge Eating Behaviors (BES)

The BES (Gormally et al., 1982) is a sixteen-question questionnaire used to assess the presence and the level of binge eating behaviors indicative of an eating disorder. The questions are based on behavioral characteristics (e.g., the amount of food consumed) and emotional, cognitive, guilt, or shame response. Each question has three/four separate answers which are assigned a numerical value. The scoring range is from 0 to 46: no-binging for values less than or equal to 17, moderate binging for values between 18 and 26, and strong binging for values equal to or greater than 27. In this study, the BES showed good reliability (*α* = 0.90).

### Data Analysis

We ran on our data a set of basic statistical analyses, *t* for groups differences, correlation, linear regression, and also three moderation analyses to understand if the relationship between two variables is moderated by the value of a third variable. SPSS version no. 26 was used for the analyses. The data satisfied the assumptions of normality of the distribution, suitable for parametric analyses.

## Results

Table 1 shows the mean and standard deviation of FCV-19S, POMS, EDI-2, and BES. The sample in the whole was found to be no-binging (mean total BES score of 8.04, much lower than the critical value of 17), as well as no pathological alterations in mood was evidenced (total mood disturbance score – TMD of the sample: 60.08). Furthermore, the dimensions explored with EDI-2 did not show significant disease scores. These results confirmed the substantial non-pathological condition of the sample, allowing the results to be interpreted as usual dynamics in a condition of “normal” distress for the isolation due to the pandemic.

**TABLE 1:** Mean and standard deviation of FCV-19S, POMS, EDI-2, and BES.

	FCV-19 S	BD	DT	MF	B	IA	I	ID	P	BES	T	A	TI	D	C	TMD
*M*	15.90	28.30	23.50	25.80	15.80	29.10	30.90	24.60	20.90	8.04	13.70	15.10	13.10	18.90	13.10	60.00
*SD*	6.06	4.68	9.00	5.78	7.539	9.20	5.55	4.15	6.04	7.81	7.52	10.6	6.65	13.8	5.69	42.7

*FCV-19S, Fear of COVID-19 Scale; BD, Body Dissatisfaction; DT, Drive for Thinness; MF, Maturity Fears; B, Bulimia; IA, Interoceptive Awareness; I, Ineffectiveness; ID, Interpersonal Distrust; P, Perfectionism; BES, Binge Eating Scale; T, Tension; A, Anger; TI, Tiredness; D, Depression; C, Confusion; TMD, Total Mood Disturbance.*

Table 2 shows the gender differences in FCV-19S, POMS, EDI-2, and BES calculated through the *t*-test of independent samples. The results presented significant differences concerning gender in some variables: FCV-19S total score, interoceptive awareness of EDI-2, BES total score and T, TI, D, C, TMD of POMS. Specifically, women were more exposed to fear of COVID-19 showing significantly higher levels of tension, fatigue, depression and confusion and a higher total mood disturbance score than males. Furthermore, Cohen’s *d* was calculated to indicate the effect size. In detail, the effects of gender differences are larger for FVC-19S, IA, TMD (total score and several sub-scores).

**TABLE 2:** Gender differences in FCV-19S, POMS, EDI-2, and BES measured using independent samples *t*-test.

	Gender	*Mean*	*SD*	*t*	*p* (df = 467)	Cohen’s *d*	Mean difference	Std. error difference	95% confidence interval of the difference	
									Lower	Upper
FVC-19S	M	15.02	6.02	–2.98	<0.01	–0.28	–1.65	0.55	–2.75	–0.56
	F	16.68	6.00							
BD	M	28.32	4.79	0.07	0.94	<0.01	0.03	0.43	–0.82	0.88
	F	28.29	4.58							
DT	M	26.28	5.70	–1.57	0.12	–0.21	–1.09	0.69	–2.46	0.27
	F	25.50	5.84							
MF	M	22.51	8.59	1.44	0.15	0.13	0.77	0.53	–0.27	1.82
	F	24.41	9.29							
B	M	15.29	7.27	–1.57	0.12	–0.15	–1.09	0.69	–0.32	0.36
	F	16.39	7.74							
IA	M	15.29	7.27	–2.95	<0.01	–0.27	–2.49	0.84	–4.15	–0.83
	F	16.39	7.74							
I	M	27.74	9.12	–0.65	0.52	–0.06	–0.33	0.51	–1.34	0.67
	F	30.24	9.13							
ID	M	30.74	5.46	0.25	0.80	0.02	0.09	0.38	–0.65	0.85
	F	31.07	5.63							
P	M	24.69	3.88	0.54	0.59	0.05	0.30	0.55	–0.79	1.40
	F	24.59	4.38							
BES	M	7.21	7.53	–2.19	0.03	–0.20	–1.58	0.72	–2.99	–0.16
	F	8.79	8.00							
T	M	12.56	7.10	–3.31	<0.01	–0.31	–2.28	0.68	–3.63	–0.92
	F	14.84	7.73							
A	M	14.38	10.39	–1.51	0.13	–0.14	–1.49	0.98	–3.42	0.43
	F	15.88	10.84							
TI	M	12.24	6.73	–2.74	<0.01	–0.25	–1.67	0.61	–2.88	–0.47
	F	13.92	6.50							
D	M	17.03	12.53	–2.80	<0.01	–0.26	–3.56	1.27	–6.06	–1.06
	F	20.59	14.72							
C	M	17.11	7.09	–2.90	<0.01	–0.27	–1.51	0.52	–2.54	–0.49
	F	14.76	6.93							
TMD	M	12.31	5.39	–2.79	<0.01	–0.26	–10.96	3.92	–18.67	–3.25
	F	13.83	5.86							

*M, Male; F, Female; FCV-19S, Fear of COVID-19 Scale; BD, Body Dissatisfaction; DT, Drive for Thinness; MF, Maturity Fears; B, Bulimia; IA, Interoceptive Awareness; I, Ineffectiveness; ID, Interpersonal Distrust; P, Perfectionism; BES, Binge Eating Scale; T, Tension; A, Anger; TI, Tiredness; D, Depression; C, Confusion; TMD, Total Mood Disturbance.*

Pearson’s correlations are shown in Table 3. All the emotional states and the mood total score (TMD) measured with POMS, were significantly correlated with fear of COVID-19 (FCV-19S). Furthermore, the difficulty of maintaining responsible and maturity behavior (MF), bulimic behavior (B), drive to thinness (DT), difficulty in recognize and identify emotions (IA), assessed with EDI-2, up to possible binge eating behaviors (BES), were correlated with fear of COVID-19 Scale (FCV-19S). The emotional states (T, A, and D) assessed with POMS were significantly correlated with bulimic behavior (B), interoceptive awareness (IA), ineffectiveness (I) of EDI-2, and binge eating behaviors (BES).

**TABLE 3:** Pearson’s correlations between FCV-19S, POMS, EDI-2, and BES.

	T	A	TI	D	C	TMD	FCV-19S
FCV-19S	0.41**	0.31**	0.35**	0.33**	−0.15**	0.36**	1.00
EDI-2							
BD	–0.06	−0.01*	–0.04	–0.08	0.22**	–0.04	0.07
DT	0.30**	0.21**	0.36**	0.31**	−0.33**	0.29**	0.12*
MF	0.34**	0.30**	0.27**	0.48**	−0.11*	0.33**	0.28**
B	0.45**	0.37**	0.43**	0.43**	−0.28**	0.369**	0.24**
IA	0.63**	0.57**	0.58**	0.67**	−0.45**	0.60**	0.30**
I	0.34**	0.37**	0.35**	0.55**	−0.13**	0.43**	0.04
ID	0.08	<0.01	0.04	−0.11*	0.21**	0.04	0.14**
P	0.23**	0.18**	0.17**	0.23**	–0.01	0.17**	–0.03
BES	0.38**	0.33**	0.36**	0.41**	−0.32**	0.31**	0.19**

**p < 0.01; *p < 0.05.T, Tension; A, Anger; TI, Tiredness; D, Depression; C, Confusion; TMD, Total Mood Disturbance; FCV-19S, Fear of COVID-19 Scale; EDI-2, Eating Disorder Inventory-2; BD, Body Dissatisfaction; DT, Drive for Thinness; MF, Maturity Fears; B, Bulimia; IA, Interoceptive Awareness; I, Ineffectiveness; ID, Interpersonal Distrust; P, Perfectionism; BES, Binge Eating Scale.

Table 4 reports the results of the linear regression between the independent variable (FCV-19S) and dependent variables (TMD, EDI-2 total score, and BES). The results show that fear of COVID-19 appears to be a predictor of mood changes (TMD) and of disordered eating behavior (assessed by EDI-2 and BES).

**TABLE 4:** Linear model of fear COVID-19 predictor of profile mood state (TMD) and disordered eating behaviors (EDI-2 and BES).

	Standardized coefficients	*t*	*p*	95% confidence interval for B	
	Beta			Lower bound	Upper bound
BES					
Constant		4.25	<0.01	2.27	6.19
FCV-19S	0.19	4.09	<0.01	0.12	0.36
EDI-2					
Constant		38.14	<0.01	142.50	157.90
FCV-19S	0.25	5.60	<0.01	0.84	1.75
TMD					
Constant		3.84	<0.01	9.71	30.00
FCV-19S	0.36	8.31	<0.01	1.93	3.13

Three moderation analyses were conducted in which fear of COVID-19 was considered as a predictor variable, sex as a moderator variable and EDI-2, BES, and TMD as dependent variables, respectively.

These analyses revealed significant effect of fear of COVID-19:

-For eating disorders measured by EDI-2 (β = 1.10, *SE* = 0.34, *p* < 0.01) but not by sex (β = −2.10, *SE* = 8.0, *p* = 0.79). The interaction effect was also not significant (β = 0.29, *SE* = 0.46, *p* = 0.53).

-For binge eating disorders measured by BES (β = 0.27, *SE* = 0.08, *p* < 0.01) but not by Sex (β = 2.50, *SE* = 2.00, *p* = 0.20). The interaction effect was also not significant (β = −0.08, *SE* = 0.11, *p* = −0.72).

-Mood changes (TMD) (β = 2.10, *SE* = 0.44, *p* < 0.01) but not by Sex (β = −2.10, *SE* = 10, *p* = 0.83). The interaction effect was also not significant (β = 0.57, *SE* = 0.61, *p* = 0.35).

## Discussion and Conclusion

The data from this study are consistent with the current literature regarding the impact of COVID-19 on the emotional states and eating behaviors of young people.

Studies carried out before the COVID-19 period had already analyzed the link between eating behaviors and emotional states. Micanti et al. (2017) showed that eating behavior is connected to an emotional dysregulation connected to some mental dimensions: impulsivity, body image, and anxiety. Where there is an increase in this size, there could be an imbalance in the emotional regulation system. This leads the subjects to adopt dysfunctional eating behaviors (Micanti et al., 2017).

The sample of our study did not show pathological changes in eating behaviors and mood states. Specifically, results confirmed the substantial non-pathological condition of the sample, allowing the results to be interpreted as typical dynamics in a condition of distress for the isolation due to the pandemic. However, it is interesting to note how the fears related to COVID-19 increase with the increase in mood regulation and eating behaviors. Significant correlations were highlighted between the total score of mood states, some dimensions measured with EDI-2 (MF, B, DT, IA) up to possible binge eating behaviors measured with BES and fear of COVID-19.

The regression analysis of the study showed how fear associated with COVID-19 is a predictor of both mood states and eating behaviors. Therefore, a particularly traumatic event such as COVID-19 could make latent discomfort evident. In this period of pandemic, we were “bombed” with news that always associated with the virus, infections, hospitalized in intensive care units, deaths. This led the subjects to “mull over” always on the same topics, even more during the period of isolation, in which many distractions were not possible. This finding is supported by recent literature by Smith et al. (2018). In details, the authors have highlighted how rumination, a cognitive process in which repetitive thoughts about negative experiences and emotions prevail, is implicated in the psychopathology of behaviors and/or eating disorders (Smith et al., 2018).

As showed in a recent study by Sarwer et al. (2019) the impulse control plays a dominant role in eating disorders. Furthermore, this research highlighted how the dimensions of “tension” and “anger” are correlated with bulimic-type eating behaviors.

A recent study (McCuen-Wurst et al., 2018) underlined how eating disorders are associated with some psychopathological dimensions, such as particular anxiety, mood disorders. These data are in line with the results of our study because the subjects examined there were many significant correlations between mood states and eating behaviors.

Several studies report a higher rate of eating disorders in the female gender (Asarian and Geary, 2013; Taha et al., 2018). Moreover, in this study the binge eating disorder showed a gender significant difference, with prevalence in female. As regards the other variables of eating behavior evaluated with EDI-2, no significant gender differences were highlighted. On the other hand, women were more exposed to fear of COVID-19 showing greater tension, fatigue, depression, confusion and a significantly higher total mood disturbance score than males. However, although COVID-19 is found to be a predictor of both mood states and eating behaviors, the moderation analysis highlighted how the gender variable does not significantly intervene in moderating this result, differentiating these results from other previously obtained in conditions of real pathologies.

Finally, a number of important limitations need to be considered. First: it constitutes only a preliminary investigation of some interesting variables examined during the COVID-19 pandemic. Indeed, the study participants do not constitute a sample of subjects diagnosed with eating disorders, so the results are linked to the consequences of the “normal” stress due to the fear of pandemics. Second: a limitation of this study is that the analysis doesn’t provide additional comparisons of the three variables altogether with SEM models, which was not in the scope of this study. Third: another important limitation is the lack of available comparisons between pre and post COVID-19 experiences. Indeed, the new findings added by this research concern emotional states and eating behavior during the COVID-19 conditions. This limit is important to keep in mind, as it is not possible to know if the conditions found in our sample would have been highlighted even in the absence of a COVID-19 pandemic. Acknowledging the study’s limitations, we note that future longitudinal clinical research could improve this preliminary knowledge. More longitudinal research is therefore needed to provide a deeper understanding of the long-term impact of COVID-19 conditions on eating disorders and mood states, also in non-pathological samples.

The data gives indications relating to the possibility that targeted preventive interventions can be carried out aimed at certain categories of subjects, such as university students, regardless of gender. For example, it would be important to provide support regarding the variables where difficulties have been identified (e.g., the emotional aspects more correlated with the fear of pandemics, as the difficulty in recognize and identify emotions), to avoid that, with a particularly stressful event, more serious discomfort can be revealed.

Considering that alterations in the emotional profile and maladaptive eating behaviors respond well to supportive interventions, we can suggest the need to implement strategies against psychological distress during the health emergency by COVID-19, and to design psycho-educational interventions, in young people too, aimed at modifying the lifestyle and behaviors at risk of mental disorder in favor of health-oriented behaviors. Moreover, as has been suggested (Talevi et al., 2020), evidence about these preventive aims is needed for the future.

## Data Availability Statement

The raw data supporting the conclusions of this article will be made available by the authors, without undue reservation.

## Ethics Statement

The studies involving human participants were reviewed and approved by the Ethic Committee of the Department of Educational Sciences of the University of Catania (Italy).

## Author Contributions

CD, DC, and SD: conceptualization. CD, FS, MP, and ZH: methodology. FS: validation. FS and ZH: formal analysis. MP: investigation. DC: data curation. CD, MP, and FS: writing-original draft preparation. CD, DC, and MP: writing-review and editing. RLC and SD: visualization and supervision. RLC and CD: funding acquisition. All authors contributed to the article and approved the submitted version.

## Conflict of Interest

The authors declare that the research was conducted in the absence of any commercial or financial relationships that could be construed as a potential conflict of interest.
